# The Combined Effects of Cannabis, Methamphetamine, and HIV on Neurocognition

**DOI:** 10.3390/v15030674

**Published:** 2023-03-03

**Authors:** Jeffrey M. Rogers, Jennifer E. Iudicello, Maria Cecilia G. Marcondes, Erin E. Morgan, Mariana Cherner, Ronald J. Ellis, Scott L. Letendre, Robert K. Heaton, Igor Grant

**Affiliations:** 1 San Diego State University/University of California San Diego Joint Doctoral Program in Clinical Psychology, San Diego, CA 92120, USA; 2 Department of Psychiatry, University of California San Diego, San Diego, CA 92093, USA; 3 San Diego Biomedical Research Institute, San Diego, CA 92121, USA; 4 Department of Neurosciences, University of California San Diego, San Diego, CA 92093, USA; 5 Department of Medicine, University of California San Diego, San Diego, CA 92093, USA

**Keywords:** HIV, methamphetamine, cannabis, polysubstance use, substance use disorder, cognition

## Abstract

Objective: Methamphetamine and cannabis are two widely used substances among people living with HIV (PLWH). Whereas methamphetamine use has been found to worsen HIV-associated neurocognitive impairment, the effects of combined cannabis and methamphetamine use disorder on neurocognition in PLWH are not understood. In the present study, we aimed to determine the influence of these substance use disorders on neurocognition in PLWH and to explore if methamphetamine-cannabis effects interacted with HIV status. Method and Participants: After completing a comprehensive neurobehavioral assessment, PLWH (*n* = 472) were stratified by lifetime methamphetamine (M−/M+) and cannabis (C−/C+) DSM-IV abuse/dependence disorder into four groups: M−C− (*n* = 187), M−C+ (*n* = 68), M+C−, (*n* = 82), and M+C+ (*n* = 135). Group differences in global and domain neurocognitive performances and impairment were examined using multiple linear and logistic regression, respectively, while holding constant other covariates that were associated with study groups and/or cognition. Data from participants without HIV (*n* = 423) were added, and mixed-effect models were used to examine possible interactions between HIV and substance use disorders on neurocognition. Results: Compared with M+C+, M+C− performed worse on measures of executive functions, learning, memory, and working memory and were more likely to be classified as impaired in those domains. M−C− performed better than M+C+ on measures of learning and memory but worse than M−C+ on measures of executive functions, learning, memory, and working memory. Detectable plasma HIV RNA and nadir CD4 < 200 were associated with lower overall neurocognitive performance, and these effects were greater for M+C+ compared with M−C−. Conclusions: In PLWH, lifetime methamphetamine use disorder and both current and legacy markers of HIV disease severity are associated with worse neurocognitive outcomes. There was no evidence of an HIV × M+ interaction across groups, but neurocognition was most impacted by HIV among those with polysubstance use disorder (M+C+). Better performance by C+ groups is consistent with findings from preclinical studies that cannabis use may protect against methamphetamine’s deleterious effects.

## 1. Introduction

Despite advances in HIV treatment improving life expectancy [1] and showing possible efficacy for improving neurocognitive outcomes [2], neurocognitive impairment (NCI) continues to affect 25–50% of people living with HIV [3,4,5,6]. NCI has been associated with functional impairments [7,8,9,10,11] and may increase the risk of more rapid HIV progression and earlier mortality [12,13]. Understanding the factors that increase the risk for and/or exacerbate neurocognitive impairment in people living with HIV (PLWH) is of particular clinical importance.

Studies have shown that there are complex relationships between HIV treatment, neurocognitive impairment, and substance use: HIV progression and substance use are independently associated with neurocognitive impairment [7,9,14,15,16,17], and substance use is associated with worse adherence to combination antiretroviral therapy (cART) regimen [18,19]. Additionally, cART regimen interference is associated with increased NCI risk [20], which itself is associated with poorer medication adherence and more clinically severe substance use [21,22,23]. Methamphetamine and cannabis are two substances of particular relevance, as cannabis use prevalence in PLWH is three times as high as in the general population [24], and methamphetamine use is closely related to HIV infection risk [25,26,27]. Overall, use of these substances together is quite prevalent in the US, as past-month self-report data from the National Survey on Drug Use and Health (NSDUH) indicate that the majority (62.2%) of people who used methamphetamine also used cannabis [28].

The evidence linking methamphetamine use to neurocognitive deficits is robust, with lower relative performance and greater rates of impairment being observed in memory, executive functioning, information-processing speed, and visuospatial abilities [29,30,31,32,33]. 

In studies of PLWH, methamphetamine use has been associated with a greater likelihood of displaying neurocognitive impairment [15,17,34] and loss of independent functioning [14,35]. 

The data on cannabis, neurocognition, and HIV are mixed. Regarding the effects of cannabis, independent of HIV disease, an early meta-analytic study suggested that long-term cannabis use was not associated with general neurocognitive impairment except for a small effect on memory [36]. More recent evidence and meta-analytic efforts suggest that after removing the influence of acute and/or brief residual effects, there were no significant differences in neurocognition attributable to histories of cannabis use [37,38]. In PLWH, a review conducted between 2000 and 2013 found that cannabis use was associated with poorer memory task performance in a subset of examined studies [39]. More recent studies suggest that cannabis use does not exacerbate neurocognitive performance deficits resulting from HIV disease progression [40], and significant lifetime cannabis exposure has even been linked to a lower likelihood of neurocognitive impairment in PLWH [41].

Despite the high prevalence at which methamphetamine and cannabis use co-occur, the effects of combined methamphetamine and cannabis use in HIV have received less attention, though it is possible that anti-inflammatory and other neuroprotective mechanisms attributable to cannabis might attenuate the additive injury posed by methamphetamine and HIV [32]. In a non-methamphetamine-exposed, cannabis-using PLWH cohort, there was evidence of less neuro- and systemic inflammation, and possibly better neurocognitive performance [41,42]. 

Preclinical and clinical studies have identified neurocognitive deficits resulting from HIV disease progression and methamphetamine use, but the evidence is mixed regarding cannabis effects and is especially limited in understanding how cannabis interacts with methamphetamine and HIV disease processes. The aims of the present study were to (1) examine the impact of lifetime methamphetamine and/or cannabis use disorder histories and characteristics on neurocognitive outcomes in people living with HIV and (2) determine if the patterns or severity of neurocognitive effects of methamphetamine and cannabis use histories differed in PLWH vs. those without HIV.

## 2. Method

### 2.1. Sample

Participants included 472 PLWH enrolled in NIH-funded research studies (see Funding section for details) conducted at the UCSD HIV Neurobehavioral Research Program (HNRP). Participants provided written informed consent to undergo study procedures, which were approved by the UCSD Institutional Review Board. Participants’ data were included in this secondary analysis if they completed a comprehensive neuromedical, neurocognitive, psychiatric, and substance use assessment. Stratifying participants by the presence or absence of lifetime methamphetamine (M+/M−) and cannabis (C+/C−) use, abuse, or dependence diagnoses [43] yielded four study groups: M−C− (*n* = 187), M−C+ (*n* = 68), M+C− (*n* = 82), and M+C+ (*n* = 135). 

Participants were excluded from analyses according to the following criteria: (1) Participants presented for their assessment with a positive breathalyzer test for alcohol or urine drug screen for substances other than methamphetamine or cannabis. (2) Participants met the criteria for DSM-IV alcohol or other (non-cannabis, non-METH) substance abuse/dependence within one year of assessment. (3) Presence of any known active major neurological (e.g., seizure, stroke) or psychiatric (e.g., psychosis) conditions, learning disabilities, or dementia diagnosis that may confound their performance on neurocognitive measures. (4) Wide Range Achievement Test-4 (WRAT-4; [44]) reading subtest standard scores < 80.

Also included for initial modeling of the interaction between HIV status and substance use disorder group contrasts were data from 423 people living without HIV (PLWoH) who completed comprehensive assessments as part of their participation in UCSD HNRP-associated NIH-funded studies, and whose data have been analyzed and reported [45]. These data were subject to the same inclusion/exclusion criteria and the same substance use disorder group stratification paradigm. Descriptive statistics for the whole sample, split by PLWH and PLWoH, are provided in Supplementary Table S1, and stratification of the PLWoH sample by substance use disorder group is provided in Supplementary Table S2.

### 2.2. Measures

Data were collected using standardized neuromedical, neurocognitive, and psychiatric evaluations. A medical history interview was used to assess for current and past medical conditions (e.g., Hepatitis C Virus [HCV], diabetes, hypertension, hyperlipidemia). A breathalyzer assessment was used to screen for recent alcohol use, and blood and urine specimens were collected for routine clinical labs, diagnostic (e.g., HIV, HCV) tests, and urine toxicology screening. No participants had a positive breathalyzer test on the morning of the evaluation. 

#### 2.2.1. HIV Disease Characteristics

Antiretroviral (ARV) usage history was measured using a structured, clinician-administered questionnaire, and the duration of HIV disease was measured as the intervening time between the first positive HIV test and the date of the neuromedical examination. HIV-infection status was determined by rapid vertical flow HBc/HIV/HCV serum antibody test (Miriad POU+, MedMira, Halifax, NS, Canada). HBc data were not available at the time of this study, with the exception of a subset of participants (*n* = 92, 19.5%). Of the available data, only 5% were HBV+. Complete blood counts, rapid plasma reagin, hepatitis C virus antibody, and CD4+ T cells were measured using routine clinical chemistry panels. Levels of HIV viral load in plasma and CSF were measured using reverse transcriptase polymerase chain reaction (Amplicor, Roche Diagnostics, Indianapolis, IN, USA), with a lower limit of quantitation (LLQ) of 50 copies/mL above which point HIV viral load was categorized as detectable in plasma. Because participants’ data were derived from multiple studies, some assays used to measure plasma and CSF HIV had LLQs of 20 copies/mL–40 copies/mL. In these cases, HIV viral load was dichotomized as undetectable if the viral load value fell below the LLQ of the given assay. RNA polymerase chain reaction kits were standardized for the detection of HIV from CSF.

#### 2.2.2. Psychiatric and Substance Use History

Criteria for current and lifetime substance use (i.e., DSM-IV Substance Abuse/Dependence) and mood disorders were assessed using the Composite International Diagnostic Interview [46]. Lifetime histories of cannabis [47] and methamphetamine [48] use were detailed using a semi-structured Timeline Follow-Back substance use interview. Variables derived from the Timeline Follow-Back included the following: age at first use, years since last use, age at first and the most recent cannabis/methamphetamine use disorder diagnosis. The Beck Depression Inventory—2nd version [49] was used to assess participants’ current depressive symptoms.

#### 2.2.3. Neurocognitive Performance

Participants completed a standardized battery of tests to evaluate neurocognitive functioning, which included an estimate of premorbid verbal IQ (i.e., Wide Range Achievement Test-4 [WRAT-4] Reading subtest). The battery included 14 tests assessing 7 domains relevant to methamphetamine and/or cannabis [41,48] including verbal fluency, information-processing speed, executive functions, learning, memory, working memory, and motor skills (for a list of tests, see [4]). Raw scores from individual tests were converted to T scores (Mean = 50; SD = 10), which were demographically adjusted for age, education, sex, and race/ethnicity as appropriate based on published normative samples [50,51,52]. Individual test T scores were averaged within the domain to obtain domain neurocognitive T scores and together to obtain a global neurocognitive T score. Global and domain neurocognitive T scores were used to assess neurocognitive performance across the study groups, where higher values indicated better performance.

#### 2.2.4. Neurocognitive Impairment (NCI)

Demographically corrected T scores from individual tests were also converted into deficit scores, ranging from 0 (T score > 39, no impairment) to 5 (T score < 20, severe impairment) and averaged to create domain deficit scores (DDS) and a global deficit score (GDS), which were used as outcome variables in analyses. To classify global impairment, we used a cutoff of greater than or equal to 0.5, a score that represents performance that is at least mildly impaired on at least half of the tests in the battery [52,53]; domain impairment DDS cutoff is >0.5.

### 2.3. Data Analysis

All analyses were conducted using R (version 4.2.1). For neuromedical and psychiatric variables listed in we used Kruskal-Wallis analysis of variance for continuous variables and chi-square tests for categorical variables to determine which variables differed between substance use groups. For those that were found to significantly differ, we generated Pearson correlation matrices (point-biserial correlations for categorical variables) with global and neurocognitive domain T scores to determine which variables warranted inclusion as covariates in subsequent modeling procedures. Current depressive symptoms (BDI-II Total Score), detectable plasma HIV RNA, nadir CD4+ T-cell count, current antiretroviral therapy status, estimated premorbid verbal IQ, metabolic syndrome, and lifetime alcohol use disorder, cocaine use disorder, and opioid use disorder were observed to significantly correlate with neurocognitive outcomes and were, therefore, included as covariates in all inferential models. Covariates were subsequently trimmed from individual models if they did not significantly improve model fit, as evidenced by likelihood ratio tests and descriptive fit indices (Akaike & Bayesian Information Criteria).

Multiple linear regression models of global and domain neurocognitive T scores were used to examine differences in performance across groups. Substance use group contrasts were determined from two primary comparisons of interest (i.e., M+C+ vs. M+C− and M+C+ vs. M−C−). We were initially interested in determining how people with both lifetime methamphetamine and cannabis use disorder differed from those with lifetime methamphetamine use disorder and from those with neither use disorder. Additionally, to examine whether lifetime cannabis use disorder was associated with neurocognitive performance for those with no methamphetamine use history, we compared M−C+ and M−C−. The same strategy was applied to multiple logistic models to examine if differences in impairment rates could be attributed to substance use disorder groups. C+ and M+ subgroup analyses were conducted to examine whether group differences in lifetime substance use characteristics influenced results. Variance inflation factors were examined for all models to ensure that multiple collinearities did not inflate model error, and where applicable, all models were estimated using robust standard error procedures [54].

Pooling data with a sample of PLWoH analyzed elsewhere [45], we used generalized linear mixed-effect (GLMER) models to examine the association between HIV status (PLWH vs. PLWoH) and neurocognitive performance/impairment across domains, and whether these associations differed by substance use group. Neurocognitive performance (i.e., T scores) and impairment (i.e., dichotomous Y/N modeled with binomial distribution and logit link function) were used as response variables. Further PLWH subgroup models were used to test for interactions between HIV disease characteristics and substance use group contrasts. The primary explanatory variables of interest were consistent with those used in multiple regression models, with additional examination of interactions between substance use group contrasts and HIV disease characteristic variables (i.e., detectable plasma HIV RNA, nadir CD4+ T-cell count, antiretroviral therapy (ART) use). Unsuppressed (detectable in plasma) HIV RNA and nadir CD4+ counts lower than 200 cells/mm^3^ were used as categorical explanatory variables, as these cut-off points have been shown to meaningfully distinguish risk for neurocognitive impairment in a large clinical study of people living with HIV [4]. Explanatory variables were initially entered as fixed-effect covariates and were retained if chi-square model comparison tests indicated that they significantly improved the model fit. Models were tested for overdispersion by comparing Pearson residuals extracted from each model with a chi-square distribution with the same degrees of freedom. Akaike (AIC) and Bayesian information criteria (BIC) were used to evaluate the goodness of model fit, and intraclass correlation coefficients (ICCs) were used to estimate the proportion of variance accounted for by model random effects [55]. The “lme4” R package used for this analysis employs adaptive Gauss–Hermite quadrature for maximum likelihood approximation [56].

## 3. Results

### 3.1. Participant Characteristics

Table 1 provides participants’ demographic, medical comorbidity, and psychiatric characteristics split by the substance use group. The sample of PLWH consisted of 472 participants who were on average 45.6 ± 11.5 years of age, majority male (*n* = 408, 86.4%), non-Hispanic white (*n* = 286, 60.6%), and educated (13.9 ± 2.5 years). Hypertension (*n* = 153, 32.5%) and hyperlipidemia (*n* = 135, 28.7%) were the most prevalent medical comorbidities, and hepatitis C infection was the only major medical comorbidity to significantly differ between groups, being most prevalent in the M+C+ group (*p* < 0.001). BDI-II scores and the prevalence of current major depressive disorder significantly differed between groups. M−C+ and M−C− displayed significantly lower current depressive symptom scores than the M+ groups (*p* < 0.001), but M+C− displayed a lower base rate of current major depressive disorder diagnoses (*p* = 0.045) than the other groups. The groups also significantly differed in the frequency of detectable plasma HIV RNA (unsuppressed viral load; *p* = 0.014), with M−C+ having a lower frequency than the other three groups.

Group and overall sample substance use characteristics are provided in Table 2. Lifetime DSM-4 abuse/dependence diagnoses at least five years removed from the data collection significantly differed between groups for alcohol (*p* < 0.001), cocaine (*p* < 0.001), and opioids (*p* < 0.001). M+C+ displayed higher rates of lifetime alcohol, cocaine, and opioid use disorder diagnoses than all other groups, and M−C+ displayed higher rates of lifetime alcohol and cocaine use disorder, compared with M+C− and M−C−. Between cannabis use disorder groups, M+C+ displayed more days since the most recent cannabis use epoch and younger age at first cannabis use disorder diagnosis based on the history provided. Methamphetamine use disorder groups were statistically equivalent for all methamphetamine use characteristic variables. 

### 3.2. Neurocognitive Performance

**Figure 1:**
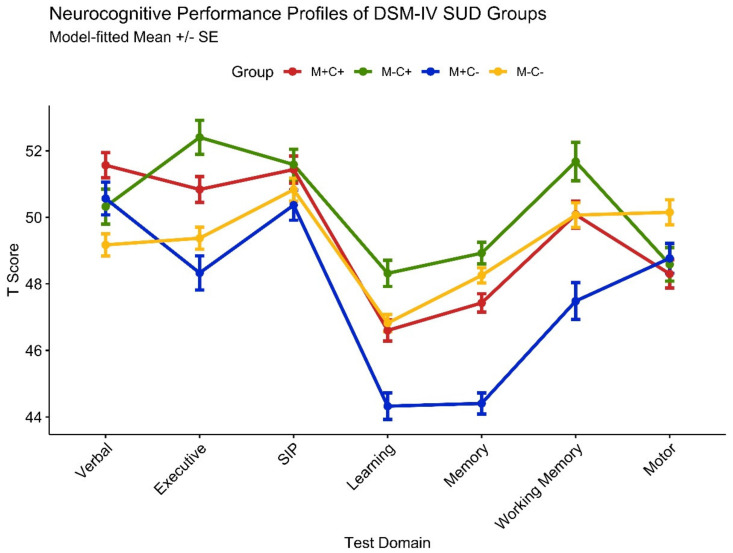
Profile plot of domain T score–predicted values in 472 PLWH from generalized linear regression models, controlled for medical comorbidities, HIV disease/treatment characteristics, current depressive symptoms, estimated premorbid verbal IQ, and other lifetime substance use. Groups represent lifetime DSM-IV substance abuse/dependence diagnoses for cannabis (C+/C−) and methamphetamine (M+/M−).

Neurocognitive domain performance (i.e., T scores) model results for substance use group contrasts are provided in the top half of Table 3. A profile plot of domain T scores is displayed in Figure 1. M+C− displayed lower performance relative to M+C+ on measures of executive functions (M_diff_ = −3.71), learning (M_diff_ = −3.95), memory (M_diff_ = −5.58), and working memory (M_diff_ = −4.05). M−C− performed worse than M+C+ on measures of verbal fluency (M_diff_ = −3.64) but performed better on measures of learning (M_diff_ = 3.46) and memory (M_diff_ = 5.19). M−C− performed worse than M−C+ on measures of executive function (M_diff_ = −3.90), learning (M_diff_ = −3.32), memory (M_diff_ = −3.38), and working memory (M_diff_ = −3.38). No group differences were observed on the global index or for information-processing speed and motor domains.

Neurocognitive domain performance model results for HIV disease characteristics are provided in the top half of Table 4. Detectable plasma HIV RNA was associated with lower memory performance (M_diff_ = −1.91). Low nadir CD4 T-cell count was associated with lower global (M_diff_ = −1.27), information-processing speed (M_diff_ = −1.82), and motor (M_diff_ = −2.81) performance. Substance use subgroup analyses indicated that cannabis and methamphetamine use characteristics (i.e., age at first use/disorder, recency of use/disorder, estimated total quantity used, estimated total duration of use) were not significantly related to the neurocognitive performance.

Initial mixed-effect modeling of the interaction between the substance use disorder group and HIV status (PLWH vs. PLWoH) on neurocognitive performance resulted in no significant interactions. Description of a model examining the main effects of the substance use disorder group and HIV disease characteristics on neurocognitive performance is provided in Supplementary Table S3. Interaction effects are displayed in Figure 3. Person-level random effects (ICC = 0.40) and neurocognitive domain-level random effects (ICC = 0.03) accounted for approximately 40% and 3% of T score variance. Controlling for premorbid verbal IQ, current depressive symptoms, metabolic syndrome, and current antiretroviral therapy use, M+C− displayed lower performance across neurocognitive score profiles than those of M+C+ (*β* = −2.23) and likewise for M−C− compared with M−C+ (*β* = −1.15). Independent of these substance use group effects, detectable plasma HIV RNA (*β* = −1.85) and low nadir CD4 T-cell counts (*β* = −1.07) were also associated with lower performance across neurocognitive score profiles. There was a significant interaction between substance use group contrasts and HIV plasma RNA detectability (see Figure 3), such that detectable HIV RNA was associated with lower performance in M+C+ compared with M−C− (*β* = 4.38, *p* = 0.011).

### 3.3. Neurocognitive Impairment

**Figure 2:**
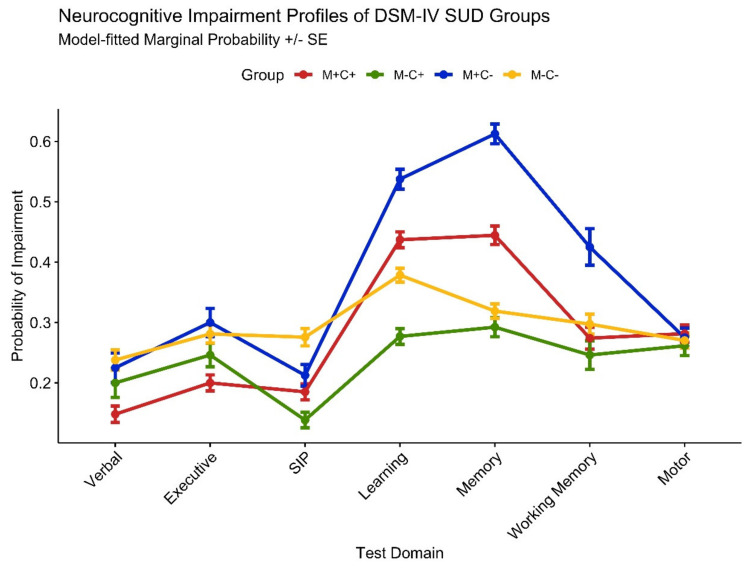
Profile plot of predicted probability of domain impairments in PLWH (*n* = 472) from binomial regression models, controlled for medical comorbidities, HIV disease/treatment characteristics, current depressive symptoms, estimated premorbid verbal IQ, and other lifetime substance use. Groups represent lifetime DSM-IV substance abuse/dependence diagnoses for cannabis (C+/C−) and methamphetamine (M+/M−).

Neurocognitive domain impairment model results for substance use group contrasts are provided in the bottom half of Table 3. A profile plot of domain impairment probability fitted values from binomial regression models is displayed in Figure 2. Higher likelihood of impairment among M+C− participants was particularly evident in their greater odds of learning (OR = 2.93), memory (OR = 5.24), and working memory (OR = 2.48) impairment compared with the M+C+ group. M−C− was significantly less likely to display learning (OR = 0.29) and memory (OR = 0.17) impairment compared with M+C+, but they were also more likely than M−C+ to display learning (OR = 3.06) and memory (OR = 2.70) impairment.

Neurocognitive domain impairment model results for HIV disease characteristics are provided in the bottom half of Table 4. Low nadir CD4 T-cell counts were associated with greater odds of motor impairment (OR = 1.61), and detectable plasma HIV RNA was not significantly associated with global or domain neurocognitive impairment. Substance use subgroup analyses indicated that cannabis and methamphetamine use characteristics (i.e., age at first use/disorder, recency of use/disorder, estimated total quantity used, estimated total duration of use) were not significantly related to the likelihood of displaying neurocognitive impairment.

**Figure 3:**
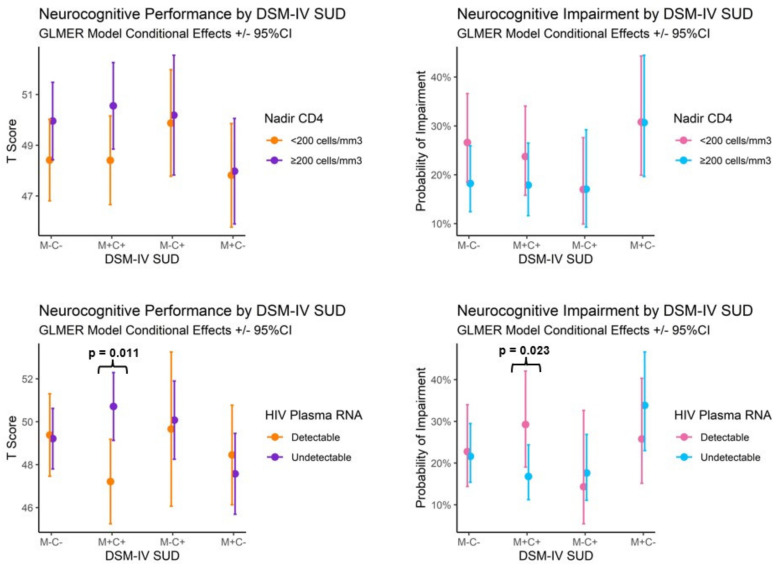
GLMER model interaction (conditional) effects estimates and 95% confidence intervals from generalized linear mixed-effect regression (GLMER) models examining overall neurocognitive performance (T scores, left) and rates of impairment (right) in PLWH (*n* = 472).

Initial modeling of the interaction between the substance use disorder group and HIV status (PLWH vs. PLWoH) resulted in no significant interactions. Description of the model examining the main effects of the substance use disorder group and HIV disease characteristics on neurocognitive impairment is provided in Supplementary Table S2. Interaction effects are displayed in Figure 3. Person-level random effects (ICC = 0.31) and neurocognitive domain-level random effects (ICC = 0.04) accounted for approximately 31% and 4% of the variance in the probability of neurocognitive impairment, respectively. Controlling for premorbid verbal IQ, current depressive symptoms, metabolic syndrome, and current antiretroviral therapy, M+C− was more likely than M+C+ to display neurocognitive impairment (OR = 1.72). Independent of substance use group effects, among people with HIV, low nadir CD4 T-cell counts were associated with greater odds of overall neurocognitive impairment (OR = 1.37). There was a significant interaction between substance use group contrasts and HIV plasma RNA detectability (see Figure 3), such that having detectable HIV RNA in plasma was associated with greater odds of impairment for M+C+ than M−C− participants (OR = 1.81, *p* = 0.023).

### 3.4. Temporality of Substance Use among M+C+ Participants

Because the temporality of drug use (e.g., if cannabis and methamphetamine use epochs coincided among those meeting the criteria for both substance use disorders) is conceptually relevant to the potential mechanisms underlying the substances’ combined effects on neurocognition, we examined M+C+ participants’ lifetime cannabis and methamphetamine use epochs. Substance initiation and continued use timelines indicate that methamphetamine use disorder epochs fell within cannabis use epochs for 123 (91.1%) M+C+ participants, indicating that use of the two substances was likely cotemporaneous. We conducted a sensitivity analysis, removing participants for whom cannabis and methamphetamine use was not cotemporaneous. These results did not differ statistically or descriptively from those reported here, indicating that this discrepancy in temporality for 8.9% of the sample did not influence the results.

## 4. Discussion

Our results indicate that in PLWH with a history of a methamphetamine use disorder, people without contemporaneous cannabis use disorder (M+C−) displayed worse neurocognitive performance and greater rates of impairment than those who had contemporaneous cannabis use disorder (M+C+). Differences in neurocognitive performance and impairment between M+C+ and M+C− suggest a possible neuroprotective effect of cannabis use in the context of methamphetamine use disorder and HIV infection. M+C− displayed performance deficits relative to M+C+ in the summary measures (T scores) of executive functions, learning, memory, and working memory domains. Based on the application of published normative data, M+C− showed greater rates of impairment in learning, memory, and working memory domains. This independent effect of contemporaneous cannabis use was observed while controlling for HIV disease characteristics, including viral suppression, premorbid verbal IQ, other lifetime substance use disorders, and other relevant medical comorbidities. The groups’ methamphetamine use characteristics (e.g., age of first use, age of first SUD diagnosis, estimated total grams used, etc.) were statistically equivalent and not found to be associated with group neurocognitive differences.

Both the methamphetamine use disorder without accompanying cannabis use and markers of more severe HIV disease were associated with worse neurocognitive performance and higher rates of impairment, but results from our models indicate that these factors are additive rather than multiplicative. We observed no significant interactions between M+ or C+ group comparisons and HIV disease status or characteristics, but we did observe a greater association between HIV viral load and worse neurocognition in M+C+, specifically (see Figure 3). This finding may be at least partially explained by the greater rates of other lifetime substance use disorders among M+C+, as polysubstance use in PLWH has been linked to worse neurocognitive outcomes. Non-significant interaction terms between M+C− and M−C− reinforce the notion that no HIV × M+ interaction was present, and rather polysubstance use disorder or some unobserved third variable better explains the greater impact of HIV disease on neurocognition among M+C+ participants with detectable HIV RNA in plasma.

In terms of magnitude, methamphetamine use disorder without cotemporaneous cannabis use was associated with greater neurocognitive T score differences than detectable HIV viral load (−2.23 vs. −1.85) and low CD4 counts (−2.23 vs. −1.85). Because most M+ participants were abstinent for at least one month prior to data collection and were, on average, over two years abstinent, observed effects of methamphetamine use disorder on neurocognition likely represent a conservative estimate of those who would be seen with an acute use disorder. Similar results were observed for neurocognitive impairment, with methamphetamine use disorder being associated with a greater odds ratio than low CD4 counts (1.72 vs. 1.37). These results are consistent with previous studies, which have shown adverse independent and additive NC effects of HIV disease markers and methamphetamine use [32,48,58]. An additive risk model is also consistent with the evidence, suggesting that substance dependence and HIV may affect different aspects of neurocognitive functioning [59]. The fact that HIV infection status alone was not associated with any neurocognitive measures in this study may be indicative of the effectiveness of antiretroviral treatment in improving neurocognitive outcomes for people living with HIV, as a substantial majority (81.9%) of participants utilizing an antiretroviral therapy regimen displayed undetectable HIV RNA in plasma (70.0%), and displayed CD4 T cell counts > 500.

People living with HIV who meet the criteria for lifetime cannabis use disorder displayed better neurocognitive performance and lower rates of impairment than those without lifetime substance use disorder diagnoses. Compared with M−C−, M−C+ performed better on measures of executive functions, learning, memory, and working memory, and they displayed lower odds of learning and memory impairment. Descriptively, there is some evidence to suggest that a history of cannabis use was associated with a larger positive difference in people living with HIV than what was observed in an HIV-negative sample (i.e., having displayed better performance in four domains compared with three). However, in terms of statistical significance, our results indicate that better performance displayed by people with a history of the cannabis use disorder or absent methamphetamine use disorder was not conditional upon HIV infection status, a detectable HIV viral load, or nadir CD4 counts.

The potential for a beneficial effect of cannabis use in this sample is consistent with a limited body of preclinical evidence indicating that cannabis may ameliorate methamphetamine-related damage in the central nervous system [32,60]. If cotemporaneous cannabis use is beneficially influencing neurocognitive outcomes for people with methamphetamine use disorder, current evidence suggests that it may be through Δ9-tetrahydrocannabinol action at both CB1 and CB2 receptors. CB1 activation has been suggested to be neuroprotective via the reduction in dopamine release and through the downregulation of glutamate-mediated excitotoxic cascades [61,62]. In addition, CB2 stimulation has anti-inflammatory effects that include a shift of macrophage phenotype from the proinflammatory M1 to anti-inflammatory M2 [32,60,63,64,65]. While these are plausible mechanisms whereby cannabis might exert protective effects against methamphetamine-induced neural injury, further preclinical studies are needed to clarify these interactions.

## 5. Limitations

While the results here support the notion that cannabis use might attenuate neural injury related to methamphetamine, several limitations need to be borne in mind in interpreting these data. While the large sample size, coupled with consistent assessment methods, may be seen as a strength, it remains the case that these are data from a single point in time. Repeated assessments of the same participants with the same tools would add precision and possibly demonstrate temporal trends, especially with continued methamphetamine abstinence. Additionally, participants’ substance use histories were based on self-report, and memory of use experiences years ago can be flawed. On the other hand, since we have focused here on classifying participants based on descriptions of substance use that met (or not) DSM-IV criteria for substance abuse/dependence, this raises confidence that we are examining groups that represent meaningful differences in substantial exposure to methamphetamine and/or cannabis. We used DSM-IV substance use disorder criteria in this study as a substantial portion of the data were collected prior to DSM-5 publication in 2013, precluding the examination of whether substance use disorder severity (i.e., mild, moderate, or severe) influenced the findings. Because our study’s aim was to characterize neurocognitive performance in people who use cannabis and/or methamphetamine, our sampling method deliberately excluded people with recent histories of other substance use disorders. While M+C+ contained a high proportion of other lifetime substance use disorders, results may have limited generalizability to current polysubstance use *in addition* to cannabis and methamphetamine. Additionally, because our analyses focused on people with past, not current histories of major drug use, we cannot extrapolate to what might be the acute neurocognitive effects of cannabis and methamphetamine.

Though we observed higher rates of Hepatitis C positive tests in the M+C+ group, we observed no significant association between Hepatitis C infection status and neurocognitive outcomes when covarying for it in statistical models. The literature on neurocognitive impairment resulting from Hepatitis C infection is mixed [66,67,68,69], but progression to more advanced liver disease may be a key differentiating fact in neurocognitive functioning among dually diagnosed HIV/HCV [70]. In the present sample, participants presenting with markers of at least mild liver disease were very few (*n* = 6, 0.13%), and this may, at least partially, explain why we observed no Hepatitis C–related effects.

As in any human research, unmeasured factors can contribute to group differences that we attribute here to the variables of interest. For example, unmeasured factors may motivate people to use methamphetamine or cannabis more exclusively, while other factors might lead to polysubstance use. Some reassurance is provided by the steps we took to model likely sources of confounds, like age, race/ethnicity, premorbid academic achievement, and various medical comorbidities, but such adjustments are never perfect. Of interest, the M+C+ group performed better neurocognitively than M+C−, despite having more extensive histories of abuse of other substances such as alcohol, cocaine, and opioids. When interpreting these results, it is important to note that our sample of people living with HIV was predominantly male (86.4%), and findings may not generalize to women living with HIV. We did not characterize the specific cannabis formulations used; therefore, we cannot attribute the effects seen here to specific cannabis components such as cannabidiol or tetrahydrocannabinol.

## 6. Conclusions

In our examination of substance abuse and neurocognition in people living with HIV (*n* = 472) and without HIV (*n* = 423), lifetime methamphetamine use disorder and both current and legacy markers of HIV disease severity were associated with worse neurocognitive outcomes. A combined history of a methamphetamine and cannabis use disorder does not appear to exacerbate methamphetamine-related deficits in people living with HIV. Instead, results are consistent with findings from preclinical studies showing that cannabis use may protect against methamphetamine’s deleterious effects. Profile analysis models showed that HIV seropositivity itself was not associated with worse neurocognitive performance, but markers of more advanced HIV disease or poorer viral suppression (i.e., nadir CD4 T cell < 200, HIV RNA detectable at ≥50 copies/mL) were associated with poorer neurocognitive performance. There was no evidence of an HIV × methamphetamine use disorder interaction; rather, our results support previous findings that methamphetamine use disorder and HIV may confer independent and additive risk for worse neurocognitive outcomes. The mechanisms underlying cannabinoids’ apparent effect on attenuating methamphetamine-abuse-associated neurocognitive impairment may relate to cannabinoids’ downregulation of excitotoxic signaling (e.g., modification of glutamatergic or dopaminergic excitotoxicity), specific anti-inflammatory actions, or possibly other neuroprotective mechanisms that deserve further exploration via animal and/or ex vivo models.

## Figures and Tables

**Table 1:** Demographic, medical comorbidity, and psychiatric characteristics split by substance use group and for the sample of people living with HIV (PLWH). Demographic and other characteristics of the sample of people living without HIV (PLWoH) are provided in Supplementary Table S1.

Demographic Characteristics	M+C+ (*n* = 135)	M−C+ (*n* = 68)	M+C− (*n* = 82)	M−C− (*n* = 187)	Total (*n* = 472)	*p*-Value ^a^
Age (Years)	46.3 (10.3)	46.3 (10.5)	45.5 (7.5)	44.8 (14)	45.6 (11.5)	0.384
Sex-Male, *n* (%)	121 (89.6%)	56 (82.4%)	75 (91.5%)	156 (83.4%)	408 (86.4%)	0.147
Education (Years)	13.3 (2.5)	13.6 (2.5)	14.2 (2.2)	14.4 (2.5)	13.9 (2.5)	0.003
Race/Ethnicity						0.189
White, *n* (%)	87 (64.4%)	43 (63.2%)	55 (67.1%)	101 (54.0%)	286 (60.6%)	
Black, *n* (%)	26 (19.3%)	18 (26.5%)	11 (13.4%)	43 (23.0%)	98 (20.8%)	
Hispanic, *n* (%)	16 (11.9%)	7 (10.3%)	11 (13.4%)	34 (18.2%)	68 (14.4%)	
Asian, *n* (%)	1 (0.7%)	0 (0.0%)	3 (3.7%)	3 (1.6%)	7 (1.5%)	
Other, *n* (%)	5 (3.7%)	0 (0.0%)	2 (2.4%)	6 (3.2%)	13 (2.8%)	
Sexual Orientation						0.045
Bisexual, *n* (%)	18 (13.3%)	6 (8.8%)	2 (2.5%)	15 (8.1%)	41 (8.7%)	
Heterosexual, *n* (%)	32 (23.7%)	18 (26.5%)	12 (14.8%)	39 (21.1%)	101 (21.5%)	
Homosexual, *n* (%)	85 (63.0%)	44 (64.7%)	67 (82.7%)	131 (70.8%)	327 (69.7%)	
Estimated Premorbid Verbal IQ ^b^	102 (11.3)	103.2 (10.9)	102 (11.8)	103.8 (12.5)	102.9 (11.8)	0.518
**Medical Comorbidity**						
Hypertension, *n* (%)	40 (29.6%)	22 (32.4%)	27 (33.3%)	64 (34.2%)	153 (32.5%)	0.853
Hyperlipidemia, *n* (%)	35 (25.9%)	24 (35.3%)	17 (21.0%)	59 (31.6%)	135 (28.7%)	0.168
Diabetes, *n* (%)	9 (6.7%)	11 (16.2%)	8 (9.8%)	23 (12.3%)	51 (10.8%)	0.175
Hepatitis C Infection, *n* (%)	33 (24.4%)	9 (13.2%)	14 (17.1%)	18 (9.6%)	74 (15.7%)	0.004
**Depression and Depressive Symptoms**						
Lifetime Major Depressive Disorder, *n* (%)	88 (65.2%)	37 (54.4%)	43 (52.4%)	95 (50.8%)	263 (55.7%)	0.068
Current Major Depressive Disorder, *n* (%)	23 (17.0%)	9 (13.2%)	4 (4.9%)	19 (10.2%)	55 (11.7%)	0.045
BDI-II Total Score ^c^	14.0 (11.1)	9.2 (10.4)	12.9 (11.7)	9.4 (9.6)	11.3 (10.7)	<0.001
**HIV Disease and Treatment**						
Detectable Plasma HIV RNA (≥50 copies/mL; *n*, %)	46 (35.4%)	10 (15.6%)	29 (37.7%)	49 (28.0%)	134 (30.0%)	0.014
AIDS Status, *n* (% AIDS)	67 (49.6%)	43 (63.2%)	50 (61.7%)	104 (55.9%)	264 (56.2%)	0.191
Estimated Duration of HIV Infection (years)	13.5 (9.1)	13.5 (8.9)	12.8 (8.5)	13.2 (9.9)	13.2 (9.3)	0.89
Nadir CD4+ T-Cell Count	223.5 (192.6)	230.7 (245.1)	227.5 (218.5)	244.9 (184.2)	233.7 (202)	0.263
Current CD4+ T-Cell Count	571.7 (296.8)	632.9 (364.6)	529.5 (307.8)	599.1 (283.1)	584.1 (304.8)	0.198
Antiretroviral Therapy (ART) Use, *n*, % on)	110 (81.5%)	57 (85.1%)	72 (87.8%)	145 (78.4%)	384 (81.9%)	0.268
ART Adherence ≥ 90% (%; *n* = 384)	98 (89.1%)	48 (81.4%)	61 (85.9%)	137 (93.8%)	344 (89.1%)	0.051
Months on current ART Regimen (*n* = 384)	26.5 (26.3)	30.6 (26.1)	20.6 (22.7)	25 (28.3)	25.4 (26.5)	0.032

Note. ^a^ Descriptive statistics were computed and compared using non-parametric tests: Kruskal-Wallis analysis of variance for continuous variables and non-parametric chi-square tests for categorical variables. ^b^ Determined by the reading subtest of the Wide Range Achievement Test-4th edition (WRAT-4). ^c^ BDI-II: Beck Depression Inventory-2nd edition. Medical comorbidity variables indicate the presence or absence of the conditions in participants’ medical history.

**Table 2:** Other lifetime substance use disorder (DSM-IV abuse or dependence) diagnoses, current substance use disorder diagnoses, lifetime cannabis exposure characteristics, and lifetime methamphetamine exposure characteristics split by substance use group and for the overall sample.

Substance Use Disorder	M+C+ (*n* = 135)	M−C+ (*n* = 68)	M+C− (*n* = 82)	M−C− (*n* = 187)	Total (*n* = 472)	*p*-Value ^a^
Lifetime-Alcohol	105 (77.8%)	47 (69.1%)	42 (51.2%)	56 (29.9%)	250 (53.0%)	<0.001
Lifetime-Cocaine	76 (56.3%)	19 (27.9%)	17 (20.7%)	18 (9.6%)	130 (27.5%)	<0.001
Lifetime-Opioid	16 (11.9%)	1 (1.5%)	5 (6.1%)	4 (2.1%)	26 (5.5%)	<0.001
Current-Alcohol	0 (0.0%)	0 (0.0%)	0 (0.0%)	0 (0.0%)	0 (0.0%)	NA
Current-Cocaine	0 (0.0%)	0 (0.0%)	0 (0.0%)	0 (0.0%)	0 (0.0%)	NA
Current-Opioid	0 (0.0%)	0 (0.0%)	0 (0.0%)	0 (0.0%)	0 (0.0%)	NA
** Cannabis-M (SD) **	** M+C+ (*n* = 135) **	** M−C+ (*n* = 68) **	**M+C− (*n* = 0)**	**M−C− (*n* = 0)**	** Total (*n* = 203) **	*** p * -Value **
Age of First Use	15.6 (2.3)	16.0 (2.3)			15.7 (2.3)	0.248
Days Since Last Use	353.3 (325.6)	281.1 (327)			329.1 (327)	0.026
Age of First CUD	19.9 (4.1)	21.6 (4.4)			20.5 (4.3)	0.009
Years Since CUD	13.8 (9.7)	12.1 (9.8)			13.2 (9.8)	0.285
Current CUD	8 (5.9%)	6 (8.8%)			14 (3.0%)	0.442
**Methamphetamine-M (SD)**	**M+C+ (*n* = 135)**	** M−C+ (*n* = 0) **	**M+C− (*n* = 82)**	**M−C− (*n* = 0)**	**Total (*n* = 217)**	*** p * -Value **
Age of First Use	26.1 (6.3)		26.9 (6.0)		26.4 (6.2)	0.259
Days Since Last Use	763.8 (779)		668 (735.3)		727.6 (762.5)	0.999
Age of First MUD	31.1 (6.4)		31.5 (6.1)		31.2 (6.2)	0.631
Years Since MUD	5.3 (4.9)		4.6 (4.5)		5 (4.7)	0.455
Current MUD	19 (14.1%)		6 (7.3%)		25 (5.3%)	0.131

Note. CUD = Cannabis Use Disorder; MUD = Methamphetamine Use Disorder. ^a^ Descriptive statistics were computed and compared using non-parametric tests: Kruskal-Wallis analysis of variance for continuous variables and non-parametric chi-square tests for categorical variables.

**Table 3:** A sample of people living with HIV (*n* = 472): the lifetime substance use disorder group contrast estimates from multiple linear regression models (β) and multiple logistic regression models (OR) with 95% confidence intervals.

T Score Generalized Linear Models	M+C+ vs. M+C−		M+C+ vs. M−C−		M−C+ vs. M−C−		
	β	95% CI	β	95% CI	β	95% CI	Ps-R^2^
Global	−2.09 ^a^	[−4.19, 0.01]	0.97	[−1.33, 3.28]	−1.80	[−4.08, 0.47]	0.12
Verbal Fluency	0.84	[−2.13, 3.81]	−3.64 *	[−6.90, −0.39]	0.71	[−2.50, 3.93]	0.09
Executive Functions	−3.17 *	[−6.12, −0.22]	1.49	[−1.74, 4.73]	−3.90 *	[−7.09, −0.70]	0.10
Information-Processing Speed	−0.86	[−3.65, 1.93]	−0.38	[−3.44, 2.68]	−0.86	[−3.88, 2.16]	0.09
Learning	−3.95 **	[−6.91, −0.99]	3.46 *	[0.22, 6.70]	−3.32 *	[−6.52, −0.12]	0.07
Memory	−5.58 ***	[−8.48, −2.67]	5.19 **	[2.05, 8.33]	−3.38 *	[−6.52, −0.24]	0.07
Working Memory	−4.05 *	[−7.09, −1.02]	3.21	[−0.12, 6.53]	−3.38 *	[−6.66, −0.09]	0.10
Motor	−0.12	[−3.56, 3.33]	0.98	[−2.80, 4.76]	0.88	[−2.85, 4.61]	0.07
** NC Impairment Binomial Models **	**M+C+ vs. M+C−**		**M+C+ vs. M−C−**		**M−C+ vs. M−C−**		
	** OR **	** 95% CI **	** OR **	** 95% CI **	** OR **	** 95% CI **	** Ps-R^2^**
Global	1.61 ^b^	[0.67, 3.84]	0.84	[0.31, 2.25]	1.06	[0.40, 2.84]	0.15
Verbal Fluency	1.18	[0.46, 2.99]	2.06	[0.72, 5.92]	0.87	[0.31, 2.45]	0.11
Executive Functions	1.46	[0.63, 3.38]	1.43	[0.55, 3.72]	1.03	[0.40, 2.64]	0.08
Information-Processing Speed	1.07	[0.42, 2.75]	1.21	[0.41, 3.56]	2.32	[0.74, 7.29]	0.08
Learning	2.93 **	[1.37, 6.28]	0.29 **	[0.12, 0.67]	3.06 *	[1.26, 7.43]	0.06
Memory	5.24 ***	[2.41, 11.39]	0.17 ***	[0.07, 0.40]	2.70 *	[1.12, 6.51]	0.10
Working Memory	2.48 *	[1.12, 5.50]	0.61	[0.24, 1.53]	1.76	[0.69, 4.51]	0.11
Motor	1.03	[0.44, 2.39]	0.94	[0.37, 2.39]	1.09	[0.43, 2.73]	0.05

Note: *** *p* < 0.001; ** *p* < 0.01; * *p* < 0.05. Models were estimated using heteroskedasticity-consistent standard errors [57]. Group contrast terms are orthogonal, and effects were estimated from models holding constant medical comorbidities, HIV disease/treatment characteristics, and other lifetime substance use. ^a^ β estimates are equivalent to the difference in T scores between groups (e.g., compared to M+C+, M+C− displayed [β = −2.09] lower T scores). ^b^ OR represents the odds ratio, or comparative difference in odds of displaying domain impairment (e.g., compared with M+C+, M+C− displayed 61% greater odds of global impairment). Ps-R^2^ = Pseudo R-squared.

**Table 4:** HIV disease characteristics effect estimates from multiple linear regression models (β) and multiple logistic regression models (OR) with 95% confidence intervals.

T Score Generalized Linear Models	Detectable Plasma HIV RNA		Nadir CD4 < 200 Cells/mm^3^		
	β	95% CI	β	95% CI	Ps-R^2^
Global	−1.04 ^a^	[−2.37, 0.29]	−1.27 *	[−2.38, −0.16]	0.02
Verbal	−1.23	[−3.09, 0.64]	−0.77	[−2.33, 0.78]	0.01
Executive Functions	−1.57	[−3.43, 0.28]	−0.62	[−2.17, 0.93]	0.01
Information-Processing Speed	0.27	[−1.46, 2.01]	−1.82 *	[−3.27, −0.37]	0.02
Learning	−1.73	[−3.55, 0.09]	−1.01	[−2.53, 0.51]	0.01
Memory	−1.91 *	[−3.71, −0.11]	−1.24	[−2.74, 0.27]	0.02
Working Memory	−0.66	[−2.59, 1.28]	−0.92	[−2.51, 0.68]	0.02
Motor	−0.44	[−2.59, 1.72]	−2.81 **	[−4.59, −1.04]	0.03
** NC Impairment Binomial Models **	**Detectable Plasma HIV RNA**		**Nadir CD4 < 200 cells/mm^3^**		
	** OR **	** 95% CI **	** OR **	** 95% CI **	**Ps-R^2^**
Global	1.40 ^b^	[0.84, 2.32]	1.48	[0.95, 2.31]	0.02
Verbal Fluency	1.04	[0.60, 1.80]	1.06	[0.67, 1.69]	0.00
Executive Functions	0.92	[0.55, 1.54]	1.36	[0.89, 2.10]	0.01
Information-Processing Speed	0.91	[0.53, 1.57]	1.46	[0.93, 2.30]	0.01
Learning	1.16	[0.74, 1.83]	1.02	[0.70, 1.49]	0.00
Memory	1.45	[0.92, 2.28]	0.93	[0.63, 1.37]	0.02
Working Memory	1.00	[0.62, 1.63]	1.35	[0.90, 2.03]	0.01
Motor	1.34	[0.82, 2.19]	1.61 *	[1.05, 2.46]	0.03

Note: *** *p* < 0.001; ** *p* < 0.01; * *p* < 0.05. Models were estimated using heteroskedasticity-consistent standard errors [57]. Group contrast terms are orthogonal, and effects were estimated from models holding constant medical comorbidities, HIV disease/treatment characteristics, and other lifetime substance use. ^a^ β estimates are equivalent to the difference in T scores attributable to the condition (e.g., detectable HIV viral load was associated with [β = −1.04] lower T scores). ^b^ OR represents the odds ratio, or comparative difference in odds attributable to the condition (e.g., detectable HIV viral load was associated with 40% greater odds of global impairment). Ps-R^2^ = Pseudo R-squared.

## Data Availability

A synthetic data set and statistical software syntax used to generate the results presented in this manuscript will be made available upon request.

## Supplementary Materials

The following supporting information can be downloaded at: https://www.mdpi.com/article/10.3390/v15030674/s1. Table S1: Demographic, medical comorbidity, and psychiatric characteristics of people living with HIV (PLWH) and people without HIV (PLWoH); Table S2: Demographic, medical comorbidity, and psychiatric characteristics, split by substance use group and for the overall sample of people living without HIV (PLWoH); Table S3: Model fit indices, fixed effects estimates, and random effects estimates for generalized linear mixed effect regression (GLMER) models examining overall neurocognitive performance (T scores) and rates of impairment in participants living with HIV (*n* = 472).
