# Supplementary material for: The Combined Effects of Cannabis, Methamphetamine, and HIV on Neurocognition
Source: Viruses. 2023 Mar 3;15(3):674. doi: 10.3390/v15030674 (PMC10058939; doi:10.3390/v15030674)
Supplement: Supplementary file 1 [file viruses-15-00674-s001.zip › METH+CAN+HIV+ Supplementary Table S1.pdf]

Supplementary Table S1. Demographic, medical comorbidity, and psychiatric characteristics of people living with HIV (PLWH) and people without HIV (PLWoH).

Demographic Characteristics	PLWoH (N=423)	PLWH (N=472)	Total (N=895)	p-value[a]
Age (Years)	44.6 (14.2)	45.6 (11.5)	45.1 (12.9)	0.258
Sex - Male, n (%)	304 (71.9%)	408 (86.4%)	712 (79.6%)	0.025
Education Years	13.6 (2.6)	13.9 (2.5)	13.8 (2.5)	0.04
Race/Ethnicity				0.212
White, n (%)	255 (60.3%)	286 (60.6%)	541 (60.4%)	
Black, n (%)	63 (14.9%)	98 (20.8%)	161 (18.0%)	
Hispanic, n (%)	81 (19.1%)	68 (14.4%)	149 (16.6%)	
Asian, n (%)	7 (1.7%)	7 (1.5%)	14 (1.6%)	
Other, n (%)	17 (4.0%)	13 (2.8%)	30 (3.4%)	
Sexual Orientation				< 0.001
Bisexual, n (%)	33 (7.9%)	41 (8.7%)	74 (8.3%)	
Heterosexual, n (%)	299 (71.2%)	101 (21.5%)	400 (45.0%)	
Homosexual, n (%)	88 (21.0%)	327 (69.7%)	415 (46.7%)	
Estimated Premorbid Verbal IQ[b]	102.6 (11.8)	102.9 (11.8)	102.7 (11.8)	0.882
**Medical Comorbidity**				
Hypertension, n (%)	85 (20.1%)	153 (32.5%)	238 (26.7%)	< 0.001
Hyperlipidemia, n (%)	60 (14.2%)	135 (28.7%)	195 (21.8%)	< 0.001
Diabetes, n (%)	34 (8.1%)	51 (10.8%)	85 (9.5%)	0.162
Hepatitis C Infection, n (%)	83 (19.7%)	74 (15.7%)	157 (17.6%)	0.117
**Depression & Depressive Symptoms**				
Lifetime Major Depressive Disorder, n (%)	140 (33.2%)	263 (55.7%)	403 (45.1%)	< 0.001
Current Major Depressive Disorder, n (%)	29 (6.9%)	55 (11.7%)	84 (9.4%)	0.014
BDI-II Total Score[c]	9.0 (10.1)	11.3 (10.7)	10.2 (10.5)	< 0.001
**Substance Use Disorder**				
DSM-IV Abuse/Dependence				0.959
M+C+	116 (27.4%)	135 (28.6%)	251 (28.0%)	
M-C+	59 (13.9%)	68 (14.4%)	127 (14.2%)	
M+C-	78 (18.4%)	82 (17.4%)	160 (17.9%)	
M-C-	170 (40.2%)	187 (39.6%)	357 (39.9%)	
Lifetime - Alcohol	237 (56.2%)	250 (53.0%)	487 (54.5%)	0.338
Lifetime - Cocaine	95 (22.5%)	130 (27.5%)	225 (25.2%)	0.084
Lifetime - Opioid	52 (12.3%)	26 (5.5%)	78 (8.7%)	< 0.001

Current - Alcohol	0 (0.0%)	0 (0.0%)	0 (0.0%)	NA
Current - Cocaine	0 (0.0%)	0 (0.0%)	0 (0.0%)	NA
Current - Opioid	0 (0.0%)	0 (0.0%)	0 (0.0%)	NA

Note. [a]*Descriptive statistics were computed and compared using non-parametric tests - Kruskal–Wallis analysis of variance for continuous variables and non-parametric chi-square tests for categorical variables.* [b]*Determined by the reading subtest of the Wide Range Achievement Test – 4th edition (WRAT-4).* [c]*BDI-II: Beck Depression Inventory – 2nd edition. Medical comorbidity variables indicate the presence or absence of the conditions in participants' medical history.*
