# Supplementary material for: The Combined Effects of Cannabis, Methamphetamine, and HIV on Neurocognition
Source: Viruses. 2023 Mar 3;15(3):674. doi: 10.3390/v15030674 (PMC10058939; doi:10.3390/v15030674)
Supplement: Supplementary file 1 [file viruses-15-00674-s001.zip › METH+CAN+HIV+ Supplementary Table S2.pdf]

Supplementary Table S2. Demographic, medical comorbidity, and psychiatric characteristics, split by substance use group and for the overall sample of people living without HIV (PLWoH).

	M+C+ (N=116)	M-C+ (N=59)	M+C- (N=78)	M-C- (N=170)	Total (N=423)	*p*-value[a]
**Demographic Characteristics**						
Age	43.5 (11.3)	44.8 (15.8)	43.0 (11.1)	46.1 (16.4)	44.6 (14.2)	0.431
Sex - Male	92 (79.3%)	44 (74.6%)	56 (71.8%)	112 (65.9%)	304 (71.9%)	0.094
Education Years	12.0 (2.2)	13.8 (2.5)	12.6 (2.4)	15.0 (2.2)	13.6 (2.6)	< 0.001
Estimated Premorbid Verbal IQ[b]	98.04 (9.07)	104.98 (11.77)	97.59 (11.82)	107.1 (11.44)	102.57 (11.78)	< 0.001
Ethnicity						0.824
Asian American Pacific Islander	0 (0.0%)	1 (1.7%)	2 (2.6%)	4 (2.4%)	7 (1.7%)	
Black	19 (16.4%)	11 (18.6%)	12 (15.4%)	21 (12.4%)	63 (14.9%)	
Hispanic	20 (17.2%)	11 (18.6%)	13 (16.7%)	37 (21.8%)	81 (19.1%)	
Other	3 (2.6%)	2 (3.4%)	5 (6.4%)	7 (4.1%)	17 (4.0%)	
White	74 (63.8%)	34 (57.6%)	46 (59.0%)	101 (59.4%)	255 (60.3%)	
**Medical Comorbidity**						
Hypertension	19 (16.4%)	17 (28.8%)	8 (10.3%)	41 (24.3%)	85 (20.1%)	0.016
Hyperlipidemia	15 (12.9%)	9 (15.3%)	10 (12.8%)	26 (15.4%)	60 (14.2%)	0.914
Diabetes	10 (8.6%)	7 (11.9%)	5 (6.4%)	12 (7.1%)	34 (8.1%)	0.637
Hepatitis-C Virus Infection	39 (33.6%)	8 (13.6%)	15 (19.2%)	21 (12.4%)	83 (19.7%)	< 0.001
Chronic Pulmonary Disease	18 (15.5%)	7 (11.9%)	10 (12.8%)	20 (11.8%)	55 (13.0%)	0.821
**Psychiatric Characteristics**						
Lifetime Major Depression	45 (38.8%)	24 (40.7%)	25 (32.1%)	46 (27.2%)	140 (33.2%)	0.117
Current Major Depression	9 (7.8%)	5 (8.5%)	9 (11.5%)	6 (3.5%)	29 (6.9%)	0.111
BDI-II Total Score[c]	13.45 (11.84)	5.81 (6.99)	13.78 (10.76)	4.92 (6.95)	9 (10.14)	< 0.001

Note: [a]*Descriptive statistics were computed and compared using Kruskal–Wallis analysis of variance for continuous variables and non-parametric chi-square tests for categorical variables.* [b]*Determined by the reading subtest of the Wide Range Achievement Test – 4th edition (WRAT-4).* [c]*BDI-II: Beck Depression Inventory – 2nd edition.*
