# Supplementary material for: The Combined Effects of Cannabis, Methamphetamine, and HIV on Neurocognition
Source: Viruses. 2023 Mar 3;15(3):674. doi: 10.3390/v15030674 (PMC10058939; doi:10.3390/v15030674)
Supplement: Supplementary file 1 [file viruses-15-00674-s001.zip › METH+CAN+HIV+ Supplementary Table S3.pdf]

Supplementary Table S3. Model fit indices, fixed effects estimates, and random effects estimates for generalized linear mixed effect regression (GLMER) models examining overall neurocognitive performance (T scores) and rates of impairment in participants living with HIV (n=472).

**Performance (T scores) Model - Main Effects**					**Impairment (Y/N) Model - Main Effects**				
**Type**	LMER	**AIC**		47383.22	**Type**	GLMER	**AIC**		7417.97
**Variance-Covariance**	Unstructured	**BIC**		47534.16	**Family**	Binomial	**BIC**		7562.07
		**Ps-R^2 (fixed)**		0.09	**Link**	Logit	**Ps-R^2 (fixed)**		0.09
		**Ps-R^2 (total)**		0.45			**Ps-R^2 (total)**		0.38
**Fixed Effects**	**β**	**95% CI**	**z**	**p**	**Fixed Effects**	**OR**	**95% CI**	**z**	**p**
Difference in NC Performance Profile Attributable to Condition					Difference in NC Impairment Profile Attributable to Condition				
M+C+ v. M-C-	-0.24	[-1.52, 1.04]	-0.37	0.71	M+C+ v. M-C-	0.81	[0.51, 1.31]	-0.85	0.40
M+C+ v. M+C-	-2.23	[-3.44, -1.02]	-3.61	< .001	M+C+ v. M+C-	1.72	[1.14, 2.60]	2.59	0.01
M-C+ v. M-C-	-1.15	[-2.16, -0.14]	-2.24	0.03	M-C+ v. M-C-	1.51	[0.95, 2.41]	1.73	0.08
Viral Load Detectable	-1.85	[-3.63, -0.08]	-2.04	0.04	Viral Load ≥ 50% Detectable	0.97	[0.60, 1.57]	-0.13	0.90
Nadir CD4 < 200	-1.07	[-2.10, -0.05]	-2.05	0.04	Nadir CD4 < 200	1.37	[1.02, 1.84]	2.08	0.04
Currently on ART	0.87	[-0.02, 1.77]	1.92	0.06	Currently on ART	0.85	[0.66, 1.10]	-1.25	0.21
Pre-Morbid Verbal IQ	0.12	[0.09, 0.15]	7.62	< .001	Pre-Morbid Verbal IQ	0.97	[0.96, 0.98]	-6.68	< .001
BDI-II Score	-0.08	[-0.12, -0.05]	-4.45	< .001	BDI-II Score	1.02	[1.01, 1.03]	4.02	< .001
**Random Effects**	**Group**	**ICC**	**SD**		**Random Effects**	**Group**	**ICC**	**SD**	
	Subject (n=472)	0.40	5.01			Subject (n=472)	0.31	1.22	
	NC Domain (n=8)	0.03	1.47			NC Domain (n=8)	0.04	0.44	

Note: [a]β estimates are equivalent to the difference in T scores attributable to the condition. [b]OR represents the odds ratio, or comparative difference in odds attributable to the condition. AIC = Akaike information criterion, BIC = Bayesian information criterion, Ps-R2 = Pseudo R-squared, ICC = intraclass correlation coefficient
